# Synthesis and review: African environmental processes and water-cycle dynamics

**DOI:** 10.1088/1748-9326/11/12/120206

**Published:** 2016-12-09

**Authors:** Charles Ichoku, Jimmy Adegoke

**Affiliations:** 1 Earth Sciences Division, NASA Goddard Space Flight Center, Greenbelt, MD, USA; 2 Department of Geosciences, University of Missouri, Kansas City, MO, USA

## Abstract

Africa’s vast landmass harbors a variety of physical processes that affect the environment and the water cycle. This focus issue on ‘African Environmental Processes and Water-Cycle Dynamics’ contains eight articles that address these phenomena from different but complementary perspectives. Fires used for agricultural and related purposes play a major role in land-cover change, surface albedo modifications, and smoke emission; all of which affect the environment and the water cycle in different ways. However, emissions of aerosols and trace gases are not restricted to fires, but also emanate from other natural and human activities. The African water cycle undergoes significant perturbations that are attributable to several factors, including the aforesaid environmental processes. These changes in the water cycle have produced severe drought and flooding events in recent decades that affect societal wellbeing across sub-Saharan Africa. The combined effects of the environmental processes and water-cycle dynamics affect and are affected by climate variability and can be propagated beyond the continent. Future studies should utilize the wealth of observations and modeling tools that are constantly improving to clearly elucidate the interrelationships between all of these phenomena for the benefit of society.

## 1. Introduction

The African Continent, with its massive landmass, is the only continent straddling both the northern and southern hemispheres, and extending almost equally beyond the tropics far into the subtropics of both hemispheres. Thus, it is significantly influenced by the global atmospheric circulatory systems from both hemispheres. Since atmospheric circulation drives water distribution, Africa is also substantially impacted by the global hydrological system (e.g. Folland *et al* 1986, Giannini *et al* 2003, 2008, Lu and Delworth 2005, Hoerling *et al* 2006, Caminade and Terray 2010, Dai 2011, Nicholson and Dezfuli 2013). Both the circulation and water-cycle dynamics affect a variety of natural and anthropogenic processes that take place in the African environment, which can generate feedback mechanisms that perturb the water cycle and vice versa (e.g. Charney 1975, Garratt 1993, Xue and Shukla 1993, Xue 1997, Nicholson 2000, Clark *et al* 2001, Taylor *et al* 2002, Li *et al* 2007, Lebel and Ali 2009, Ichoku *et al* 2016, Saha *et al* 2016).

Given the significant magnitudes and interaction potential between the African environmental processes and water-cycle dynamics, and their relevance to climate and society, it became important to publish a special issue in *Environmental Research Letters* (ERL) focused on this theme. Overall, eight articles were published in this focus issue on a variety of topics that touch upon the different aspects of this theme.

## 2. African environmental processes

The focus issue addresses a number of important environmental processes that have real or potential impacts in the short, medium, and/or long-term. One of such processes is biomass burning, which is mostly induced by humans in Africa as part of their agricultural and related practices, with far-reaching implications (e.g. Bird and Cali 1998, Dami *et al* 2012, Ichoku *et al* 2016). Burning affects most vegetated land-cover types on the continent, with most of it occurring in the savanna type, the result of which is the darkening of the land surface, thereby reducing its albedo, which contributes to the absorption of solar radiation and surface heating. However, on a regional scale, the overall albedo decrease due to biomass burning tends to recover to the prefire albedo levels within seven years (Gatebe *et al* 2014). The immediate result of burning is heat release and smoke emission, with the smoke aerosols, which include black carbon and primary organic aerosols affecting the atmospheric composition and interacting with solar radiation and clouds, thereby respectively heating the atmosphere and modifying the cloud microphysics (e.g. Zhang *et al* 2014). Similarly, the gaseous emissions from biomass burning, such as carbon dioxide, carbon monoxide, methane and other non-methane hydrocarbons, as well as other constituents, also contribute to the atmospheric composition and affect air quality and/or the Earth’s radiation budget. Incidentally, the source of such emissions is not limited to biomass burning, but includes other combustion sources, such as various fossil fuel burning, and the overall trend of emissions from burning is increasing (Liousse *et al* 2014).

## 3. African water cycle dynamics

The dynamics of some important components of the water cycle, particularly, precipitation, soil moisture, and evapotranspiration, were examined using a variety of satellite observations, in order to understand how they may be influenced by biomass burning (Ichoku *et al* 2016). That study, which was focused on the northern part of sub-Saharan Africa (NSSA—bounded between the Sahara and the Equator), revealed that changes in these water-cycle variables are mostly negatively correlated with those of biomass burning, although this negative correlation is most pronounced in the northern part of the region (the Sahel) when the analysis is based on full annual cycles, but becomes more noticeable in the southern (more humid) part of NSSA when only the dry season is considered in the analysis. Another study based on extensive use of meteorological datasets explored the dynamics of water availability relative to demand between 1979 and 2010, with specific emphasis on maize production in various parts of sub-Saharan Africa (Estes *et al* 2014). It found that, although there was an overall decrease in net radiation, which helped to alleviate pressure on water demand, the resulting water availability increases in the different regions were influenced by various factors, namely: reductions in actual demand in southern Africa and post-drought rainfall increases in the Sahel. However, some countries of East Africa actually experienced reduced water availability due to reduced rainfall and increased water demand, whereas ‘intra-seasonal supply variability generally increased in West and East Africa’ (Estes *et al* 2014, p1).

## 4. Societal and climate implications

The African environmental processes and water-cycle dynamics can have a variety of outcomes that affect the human society in Africa and beyond, both in the short and long term. One such major consequence can be extreme rainfall conditions, which can result in severe flooding that can lead to loss of life and livelihood (e.g. Douglas *et al* 2008). By developing a flood extent mapping technique using synthetic aperture radar (SAR) imagery from ENVISAT/ASAR and RADARSAT-2, and verifying with visible imagery from Landsat, Long *et al* (2014) address an important area of research that is directly applicable to matters of immediate societal benefit for water-related disaster relief or mitigation. Although that study was focused on the Chobe floodplain in the Caprivi region of Namibia, since the satellite missions used provide global coverage, the developed technique is applicable toward addressing similar problems wherever they may occur throughout Africa, and indeed the entire globe.

A long-term result of cumulative environmental processes and water cycle dynamics is the generation of perturbations that can contribute to climate forcing (e.g. Tegen *et al* 1996, Ichoku *et al* 2016). ‘Analysis of observed trends in African average near-surface temperature over the last five decades reveals drastic increases’ and climate projections show that further increases in temperatures and reductions in rainfall are expected over the next decades across sub-Saharan Africa; the consequences of which may include increased frequency of heat waves and fire danger days, as well as decreases in soil moisture availability (Engelbrecht *et al* 2015).

African environmental processes and water-cycle dynamics can also lead to impacts that extend beyond the continent. One of such prominent phenomena addressed in this focus issue involves the far-reaching impacts of the massive dust emissions from the Sahara desert and the surrounding arid and semi-arid regions. Hosseinpour and Wilcox (2014) conducted a study that provides observational evidence of interactions between the dust in the oceanic Saharan air layer (OSAL) over the Atlantic Ocean and the African easterly jet–African easterly wave (AEJ–AEW) system. Such interactions can produce dust radiative heating in the atmosphere, potentially resulting in the reinforcement of AEW activity (and thus favoring tropical cyclone formation) over the ocean and reduction of precipitation over the West African Monsoon (WAM) region.

## 5. Conclusions and recommendations for future research

The topic of this focus issue on ‘African Environmental Processes and Water-Cycle Dynamics’ has been addressed from different perspectives through the variety of studies published herein. Whether the origins of the environmental processes are natural (e.g. wind-blown dust emissions) or anthropogenic (e.g. human-induced biomass burning or other activities), they affect the water cycle, whose dynamics in-turn feedback into similar (or drive some new) environmental processes. For instance, dust emissions and biomass burning occur mainly under very low moisture conditions, and the emitted dust and smoke along with the energetics of their emission and transport processes can trigger radiative or microphysical interactions with clouds, leading to the enhancement, delay, or suppression of rainfall. Insufficient and excessive rainfall, both adversely impact societal wellbeing in different parts of Africa through drought and flooding, respectively. When prolonged, these adverse influences fuel and are fuelled by climate variability and change, making the situation even worse for the human population.

In spite of all the studies published in this focus issue and elsewhere, the subject matter addressed herein, particularly, the interactions between the different phenomena (environment processes, water-cycle, climate, society) are still very poorly understood, especially from the multi-disciplinary perspective. Therefore, more interdisciplinary studies are needed to further elucidate these interactions and advance knowledge in this domain. To be successful and effective, such studies require extensive amounts of data, tools, domain expertise (especially from within the African continent), and policy/funding support. Fortunately, the numerous global orbiting satellites that have operated (and/or continue to operate) during the last couple of decades have acquired (are acquiring) massive amounts of data, which the major space agencies that own them, such as United States National Aeronautic and Space Administration (NASA) and the European Space Agency (ESA), are kindly providing free of charge to the general public. Furthermore, powerful computer models and tools are becoming more and more sophisticated, and many of them can also be obtained free of charge. Thus, it is hoped that responsible African local, national, regional, and international organizations can provide the necessary support needed to develop local expertise and to accelerate the development and up-take of the much needed research to mitigate the adverse impacts of the natural and anthropogenic environmental processes, water-cycle dynamics, and climate on society.
